# Sympathetic Tone Induced by High Acoustic Tempo Requires Fast Respiration

**DOI:** 10.1371/journal.pone.0135589

**Published:** 2015-08-18

**Authors:** Ken Watanabe, Yuuki Ooishi, Makio Kashino

**Affiliations:** 1 Department of Information Processing, Interdisciplinary Graduate School of Science and Engineering, Tokyo Institute of Technology, Yokohama, Japan; 2 Human Information Science Laboratory, NTT Communication Science Laboratories, NTT Corporation, Atsugi, Japan; 3 CREST, Japan Science and Technology Agency, Atsugi, Japan; UNLV, UNITED STATES

## Abstract

Many studies have revealed the influences of music, and particularly its tempo, on the autonomic nervous system (ANS) and respiration patterns. Since there is the interaction between the ANS and the respiratory system, namely sympatho-respiratory coupling, it is possible that the effect of musical tempo on the ANS is modulated by the respiratory system. Therefore, we investigated the effects of the relationship between musical tempo and respiratory rate on the ANS. Fifty-two healthy people aged 18–35 years participated in this study. Their respiratory rates were controlled by using a silent electronic metronome and they listened to simple drum sounds with a constant tempo. We varied the respiratory rate—acoustic tempo combination. The respiratory rate was controlled at 15 or 20 cycles per minute (CPM) and the acoustic tempo was 60 or 80 beats per minute (BPM) or the environment was silent. Electrocardiograms and an elastic chest band were used to measure the heart rate and respiratory rate, respectively. The mean heart rate and heart rate variability (HRV) were regarded as indices of ANS activity. We observed a significant increase in the mean heart rate and the low (0.04–0.15 Hz) to high (0.15–0.40 Hz) frequency ratio of HRV, only when the respiratory rate was controlled at 20 CPM and the acoustic tempo was 80 BPM. We suggest that the effect of acoustic tempo on the sympathetic tone is modulated by the respiratory system.

## Introduction

Listening to music has various effects on people’s emotional and physical states. Previous studies have found that music affects physiological activities such as respiration and the autonomic nervous system (ANS). For instance, the respiratory rate approaches the musical tempo, and this change in respiratory rate occurs more strongly with musicians than with non-musicians [1, 2]. Listening to sedative music causes an increase in parasympathetic nervous activity [3], while listening to pleasurable music causes an increase in sympathetic nervous activity [4, 5].

However, there has been little work examining the individual characteristics of music (tempo, tone, rhythm or sound pressure level). Of these musical characteristics, it has been suggested that “tempo” plays a critical role as regards music-induced effects. The effects of music on human beings change with changes in tempo [2, 6, 7]. Bernardi et al. investigated the effects of various types of music on the activity of the ANS and respiratory rate, and they reported that tempo is more important than musical preference and other musical characteristics [2]. Bernardi’s group have also indicated the effect of breathing rate on the ANS, demonstrating that a slower breathing rate suppresses the chemoreflex response to both hypoxia and hypercapnia and enhances baroreflex sensitivity [8, 9].

However, these previous studies did not investigate the effect of the interaction between acoustic tempo and respiratory rate on the ANS. Sympatho-respiratory coupling [8–10] is a neural connection between the respiratory system and the rostral ventrolateral medulla (RVLM), which is the primary regulator of the sympathetic nervous system concerned with blood pressure. It is also suggested that the sound-induced sympathetic tone originates in the RVLM [10], which means that the respiratory network has the same system for inducing the sympathetic tone as the auditory system.

Therefore, it is possible that the effect of the tempo of a piece of music on the ANS is modulated by the respiratory system. However, this has remained unclear. In this study, we investigated the effects of the relationship between acoustic tempo and respiratory rate on the ANS.

## Materials and Methods

### Ethics statement

Before the experiment, participants were provided with an information sheet, which outlined the general purpose of the study and informed them that they could withdraw at any time without penalty. All methods employed in this study were approved by the Ethics and Safety Committees of NTT Communication Science Laboratories, and were in accordance with the Declaration of Helsinki.

### Participants

Fifty two healthy people aged 18–35 years participated in the physiological experiments described below. The experiments were performed in a sound-insulated listening room. The participants sat on a sofa and were encouraged to relax. The experiments were conducted between 13:00 and 18:00 h to minimize the effect of circadian rhythms.

### Sound Stimuli & Respiration Regulation

We used 5-min sound sequences consisting of simple drum sounds, each of which had a constant tempo. These stimuli were converted to analog signals with an audio interface (EDIROL UA-5, Roland, Japan) and presented through headphones. The sound pressure level (SPL) was determined by measuring the maximum A-weighted SPL of this sound in the slow mode. The participants listened to the sounds at 68 dB (A) in all the experiments and their respiratory rates were controlled by using a silent metronome displayed on a screen. We varied the acoustic tempo—respiratory rate combination.

### Physiological Measurements

Electrocardiograms (ECGs) and an elastic chest band Polyam-RESP (Nihon Santeku, Japan) were used to measure interbeat intervals (R-R intervals) and respiration, respectively, throughout the experiments. The analog data were amplified and digitized with a BIOPAC MP150 (BIOPAC System, USA). The sampling rate was 1,250 Hz for both the ECGs and respiration measurements. We measured the respiration just with the chest band to confirm the synchronization of the measured respiration with a metronome.

To calculate the R-R intervals in the ECG measurement, R-wave detection was performed with AcqKnowledge (analysis software produced by BIOPAC), and the result was visually screened to eliminate any inappropriate R-wave detection related to artifacts such as movement. The appropriately collected R-R interval data were resampled at 10 Hz by cubic spline interpolation. For heart rate (HR) analysis, these interpolated data of the R-R intervals were converted to second-by-second values and expressed as beats per minute (BPM) by dividing 60 by each value.

We then calculated heart rate variability (HRV) to estimate the magnitude of the ANS [11]. For the HRV analysis, we applied fast Fourier transformation (FFT) to this interpolated data of the R-R intervals. Prior to FFT, linear trend was removed and Hanning window was applied. The low-frequency (LF) and high-frequency (HF) components were obtained by integrating the power spectra over their respective ranges of 0.04–0.15 Hz and 0.15–0.40 Hz. The LF to HF component ratio (LF/HF ratio) was evaluated by dividing the LF power spectrum by that of the HF component. The LF/HF ratio of HRV is an index of sympathetic nerve activity [12, 13]. The magnitude of the HF component was evaluated by using the natural logarithms of the HF power (lnHF). The HF component of HRV represents RSA, which is an index of parasympathetic activity [14].

To calculate the respiratory interval, we performed peak detection using AcqKnowledge, and visually screened the result to eliminate any inappropriate peak detection related to artifacts such as movement. The appropriately collected respiratory interval data were resampled at 1 Hz by cubic spline interpolation. For a respiratory rate analysis, these interpolated data of the respiratory intervals were converted to second-by-second values and expressed as cycles per minute (CPM) by dividing 60 by each value.

### Experimental Procedure

The study consisted of three experiments, Experiment 1 (*n* = 18), Experiment 2 (*n* = 16) and Experiment 3 (*n* = 18). In these experiments, the participants were given general information about the experiment on arrival, and their written consent was obtained.

#### Experiment 1

In Experiment 1, we varied the respiratory rate—acoustic tempo combination and defined “conditions 1–4”. These conditions are shown in Table 1. The respiratory rate was controlled at 15 or 20 CPM and the acoustic tempo was 60 or 80 BPM or the environment was silent. In the baseline recording, respiratory rate was controlled at 15 CPM without sound presentation.

**Table 1:** The details of experimental conditions.

Condition number	Respiratory rate (CPM)	Acoustic tempo (BPM)
1	15	60
2	15	80
3	20	60
4	20	80
5	20	78
6	20	82

The combination of respiratory rate and acoustic tempo varies in each condition. CPM, cycles per minutes; BPM, beats per minutes.

**Fig 1:**
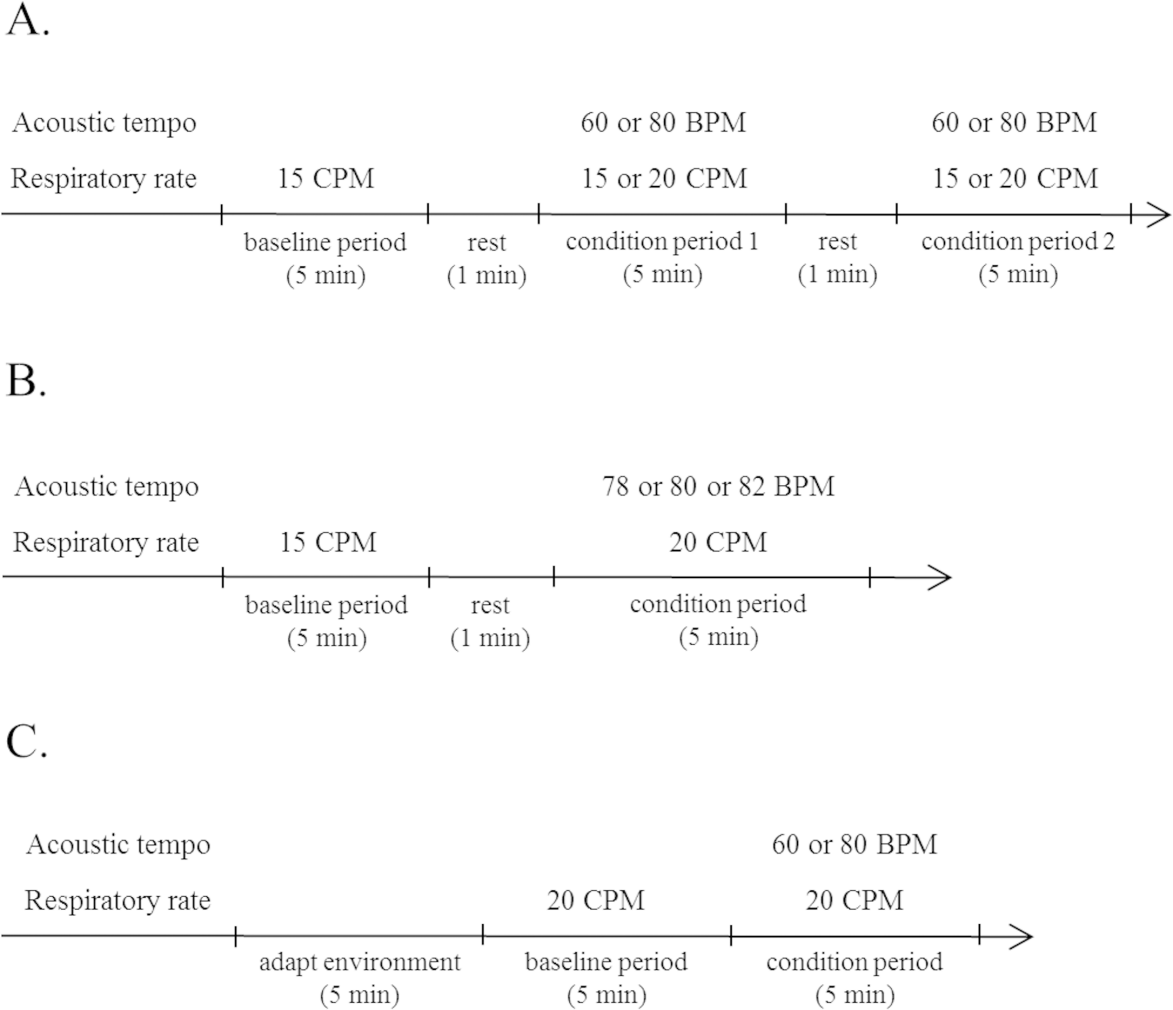
Experimental procedures. A: Procedure for Experiment 1. B: Procedure for Experiment 2. C: Procedure for Experiment 3.

The experimental procedure consisted of two sessions. The participants had a 20-min rest period after each session had finished. Prior to each session, the participants sat on a sofa while wearing headphones, and were attached to the ECG transducer electrodes and the elastic chest band for 5 min to adapt them to the experimental environment. Each session consisted of three periods: baseline period→ condition period 1→ condition period 2. Each period lasted for 5 min and the participants had a 1 min rest after each period had finished. Consequently, the participants had 4 condition periods, and conditions 1–4 were randomly assigned to these 4 periods. The 2 sessions were performed on the day participants came to the laboratory. The experimental procedures are shown in Fig 1A.

#### Experiment 2

In Experiment 2, we examined the effects of exact synchronization between the respiratory frequency and the acoustic tempo. We added the asynchronous conditions in which acoustic tempo was 78 or 82 BPM, defined as “condition 5 or 6”. These conditions are shown in Table 1. The respiratory rate was controlled at 20 cycles per minute (CPM) except for baseline recording. In the baseline recording, respiratory rate was controlled at 15 CPM without sound presentation.

The experimental procedure consisted of three sessions. The participants had a 5-min adapt environment period prior to each session and a 20-min rest period after each session had finished, as with Experiment 1. Each session consisted of two periods: baseline period→ condition period. Each period lasted for 5 min and the participants had a 1 min rest after each period had finished. Consequently, the participants had 3 condition periods, and conditions 4–6 were randomly assigned to these 3 periods. The 3 sessions were performed on the day participants came to the laboratory. The experimental procedures are shown in Fig 1B.

#### Experiment 3

In Experiment 3, we evaluated the autonomic responses in detail by calculating the HRV. We adopted conditions 3 and 4 from Experiment 1 as the sound presentation conditions, in which the respiratory rate was controlled at 20 CPM. In the baseline recording, the respiratory rate was controlled at 20 CPM without sound presentation. The experimental procedure consisted of two sessions. The participants had a 5-min adapt environment period prior to each session and a 20-min rest period after each session had finished, as with Experiment 1.

Each session consisted of two periods: baseline period→ condition period. These two periods lasted for 5 min. The order of the assignment of conditions 3 and 4 to each session was randomized. The two sessions took place on the day the participants came to the laboratory. The experimental procedures are shown in Fig 1C.

### Data Analysis and Statistical Evaluation

Time t = 0 was set as the start of each condition. The HR change was evaluated by averaging the data from t = 115 to t = 295 to exclude orienting and defense responses. The HR value averaged by this method was defined as the mean HR for each condition.

We excluded participants from the analysis whose respiratory rate was clearly irregular. The major reasons were that the participant was sleeping during the measurement or coughing too much. We excluded two individuals from Experiment 1, one individual from Experiment 2, and one individual from Experiment 3.

In Experiment 1, the respirations of 16 participants were synchronized with an electronic metronome. Since there were great differences between the mean HRs of the participants during baseline recording, we normalized the mean HR for each condition by dividing them by the mean HR of the baseline recording. The change in the mean HR with respect to the baseline was analyzed with a paired *t* test, and the difference between the normalized mean HRs for different conditions was analyzed with a two-factor repeated measures analysis of variance (ANOVA) [respiratory rate (2) x acoustic tempo (2)] as within-subjects factors. Then the difference in the mean HR was analyzed with a simple main effect test. The HRV change was evaluated by applying FFT with a 3-min Hanning window to the RR-interval data from t = 115 to t = 295. The change in the HRV with respect to the baseline was analyzed with a Wilcoxon sign rank test for the LF/HF ratio and a paired *t* test for lnHF in each session, and the difference between the normalized HRV for different conditions was analyzed with a two-factor repeated measures ANOVA [respiratory rate (2) x acoustic tempo (2)] as within-subjects factors.

In Experiment 2, the respirations of 15 participants were synchronized with an electronic metronome. We normalized the mean HR for each condition by dividing them by the mean HR of the baseline recording. The change in the mean HR with respect to the baseline was analyzed with a paired *t* test. The HRV change was evaluated by applying FFT with a 3-min Hanning window to the RR-interval data from t = 115 to t = 295. The change in the HRV with respect to the baseline was analyzed with a Wilcoxon sign rank test for the LF/HF ratio and a paired *t* test for lnHF in each session.

In Experiment 3, the respirations of 17 participants were synchronized with an electronic metronome. The HRV change was evaluated by applying FFT with 4-min Hanning window to the RR-interval data from t = 55 to t = 295. The significance of the effects of the acoustic tempo when the respiratory rate was controlled at 20 CPM on the HRV was analyzed with a Wilcoxon sign rank test for the LF/HF ratio and a paired *t* test for lnHF in each session.

The significance was defined as *p* < .05. For repeated measures ANOVA, Huynh-Feldt corrections were applied when appropriate.

## Results

### Experiment 1

#### Effect of the combination of respiratory rate and acoustic tempo on the mean HR

No significant change in respiratory rate with respect to a metronome was observed, indicating the synchronization of the respiratory rates with the electronic metronome in each condition (Figs A, B, C, and D in S1 Fig).

**Fig 2:**
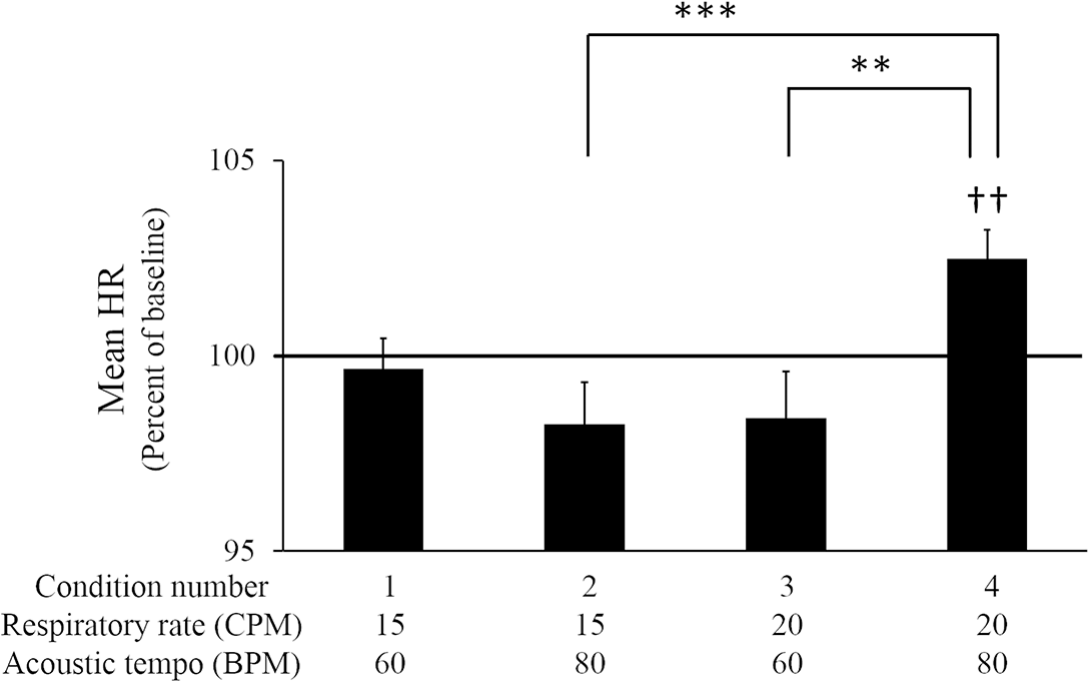
The mean HR of each condition in Experiment 1. The vertical axis indicates the normalized mean HR (percent of baseline of each session). The bar graphs and error bars represent the mean±SEM. Statistical significance is indicated as ††*p* < .01 from the baseline, **p < .01, ***p < .001 from other conditions.

The effect of the combination of respiratory rate and acoustic tempo on autonomic nerve activity was evaluated by measuring the change in the mean HR. Only the combination of a faster respiratory rate and a faster acoustic tempo caused a significant increase in the mean HR (Fig 2).

The mean HR of each condition was normalized by the mean HR of the baseline recording in each session. Of the four conditions, only condition 4 showed any change, namely a significant increase in the mean HR compared with the baseline (Cohen’s *d* = 0.83, *p* < .01). The ANOVA for the difference between the normalized mean HRs for conditions 1–4 yielded the respiratory rate×acoustic tempo interaction, *F*(1, 15) = 9.791, partial *η* ^*2*^ = 0.395, *p* < .01, and a main effect of acoustic tempo, *F*(1, 15) = 4.730, partial *η* ^*2*^ = 0.240, *p* < .05. A simple main effect test demonstrated that the normalized mean HR was significantly larger for condition 4 than for conditions 2 (*p* < .01) and 3 (*p* < .001).

**Fig 3:**
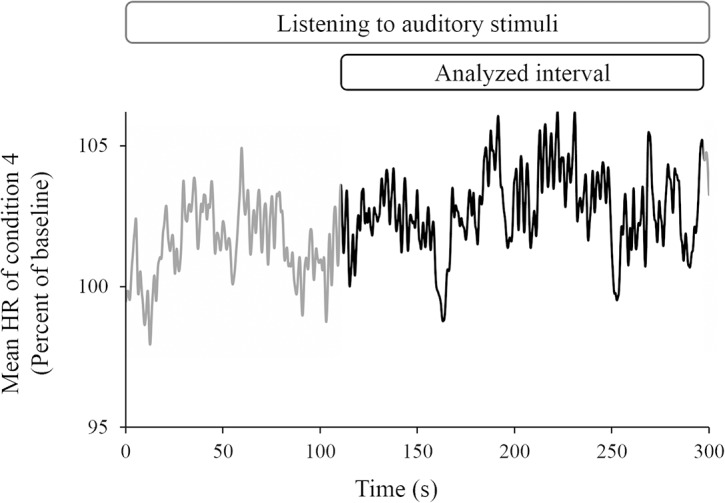
The time series data of the HR of condition 4. The data were the average HRs of all the participants. The vertical axis indicates the normalized HR (percent of baseline of condition 4). The black line indicates the analyzed interval for the mean HR from t = 115 to t = 295. The gray line indicates the interval during which the participants were listening to auditory stimuli, without any analysis.

The time series data of the HR of condition 4 are shown in Fig 3. These data were the average HRs for all participants.

**Fig 4:**
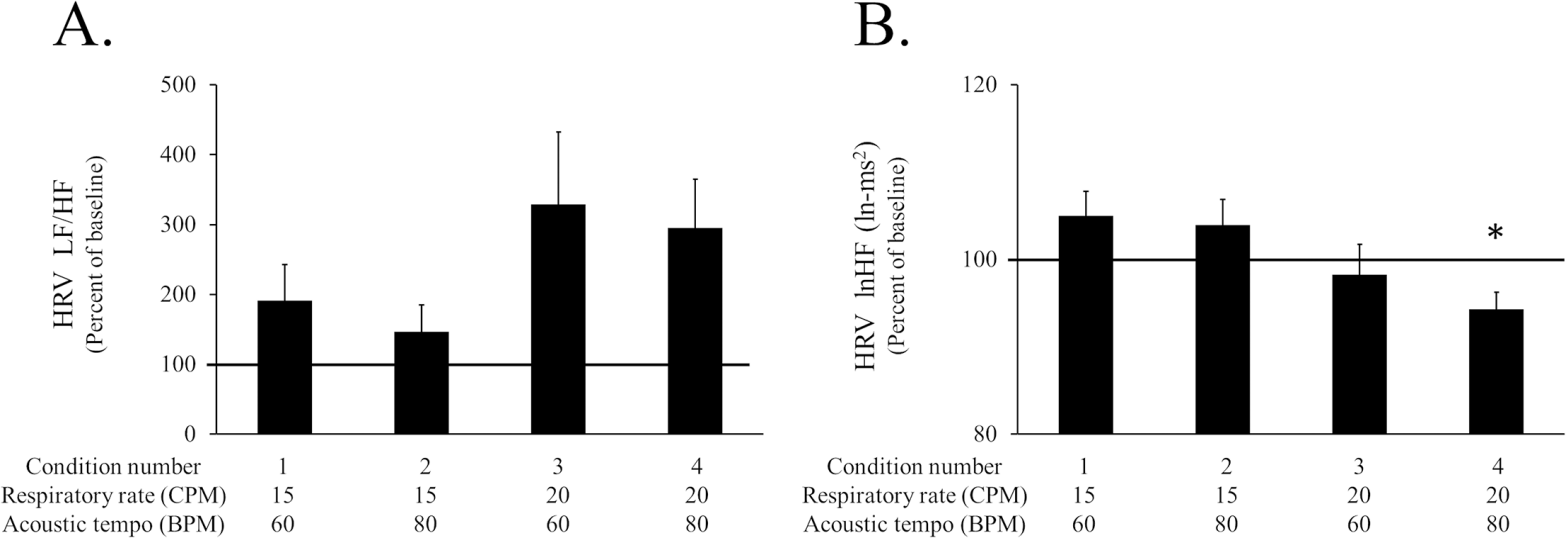
The HRV data of each session in Experiment 1. A: LF/HF of the HRV of each condition. B: lnHF of the HRV of each condition. The bar graphs and error bars represent the mean±SEM. The statistical significance is indicated as *p < .05 from the baseline.

The autonomic nerve activity was measured by the change in HRV from the baseline. A paired *t*-test demonstrated that the lnHF of the HRV was significantly smaller for conditions 4 than for the baseline condition. The ANOVA for the difference between the normalized HRV for conditions 1–4 yielded a main effect of respiratory rate, *F*(1, 15) = 11.096. However, this might be caused by the difference in respiratory rate [15]. There was no significant change in the LF/HF ratio of the HRV (Fig 4).

### Experiment 2

#### Effect of the synchronization between the respiratory frequency and the acoustic tempo

From the result of Experiment 1, there is a possibility that the synchronization between the respiratory frequency and the acoustic tempo is essential for an increase in mean HR. Since we observed a significant increase in mean HR of condition 4, we slightly changed the acoustic tempo from 80 BPM to 78 BPM and from that to 82 BPM, in which the respiratory rate was controlled at 20 CPM.

No significant change in respiratory rate with respect to the metronome was observed, indicating the synchronization of the respiratory rates and the electronic metronome in each condition (Figs E, D, and F in S1 Fig).

**Fig 5:**
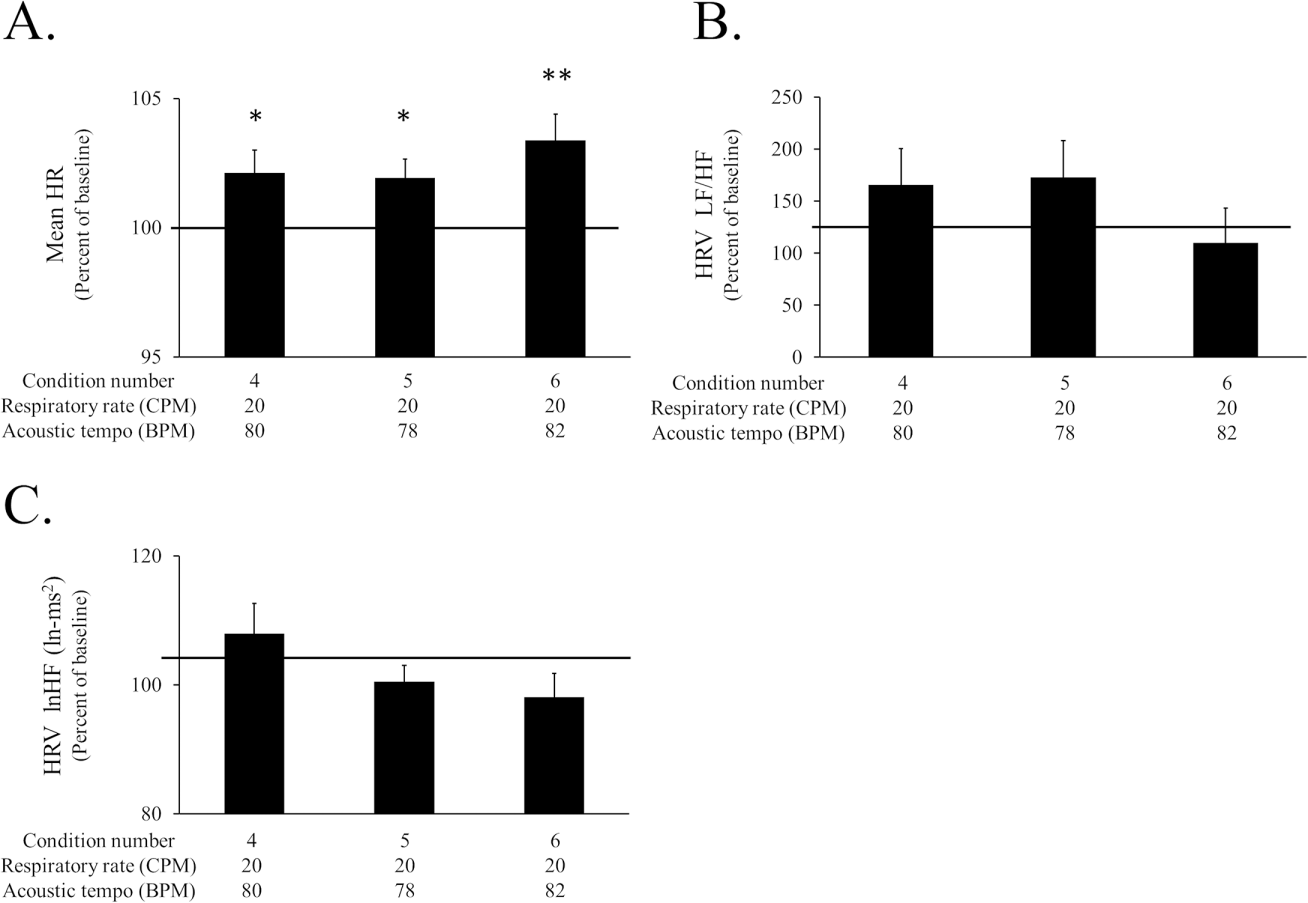
The HRV data and the mean HR of each session in Experiment 2. A: The mean HR of each condition. B: LF/HF of the HRV of each condition. C: lnHF of the HRV of each condition. The bar graphs and error bars represent the mean±SEM. The statistical significance is indicated as *p < .05, **p < .01.

The asynchronous conditions also caused a significant increase in mean HR (Fig 5A). A paired *t* test revealed that the mean HR was significantly larger for condition 4 (*t* (14) = 2.39, Cohen’s *d* = 0.14, *p* < 0.05), condition 5 (*t* (14) = 2.78, Cohen’s *d* = 0.12, *p* < 0.05) and condition 6 (*t* (14) = 3.74, Cohen’s *d* = 0.21, *p* < 0.01), respectively, than the baseline value.

The autonomic nerve activity was measured by the change in the HRV from the baseline. There was no significant change in the LF/HF ratio of the HRV and the lnHF of the HRV (Fig 5B and 5C).

### Experiment 3

#### Effect of acoustic tempo at faster respiratory rate on HRV indices

In Experiment 1, we observed that the mean HR increased only with the faster respiratory rate–faster acoustic tempo combination (20 CPM– 80 BPM). Possible mechanisms for the HR increase are enhancement of the sympathetic nerve activity, suppression of vagal nerve activity, and a combination of the two. However, we could not calculate the HRV accurately because HRV analysis is largely affected by respiratory frequency [15]. Therefore, in Experiment 3, we evaluated the autonomic responses in detail by calculating the HRV. We chose conditions 3 and 4 from Experiment 1, in which the respiratory rate was controlled at 20 CPM. In the baseline recording, the respiratory rate was controlled at 20 CPM without sound presentation.

**Fig 6:**
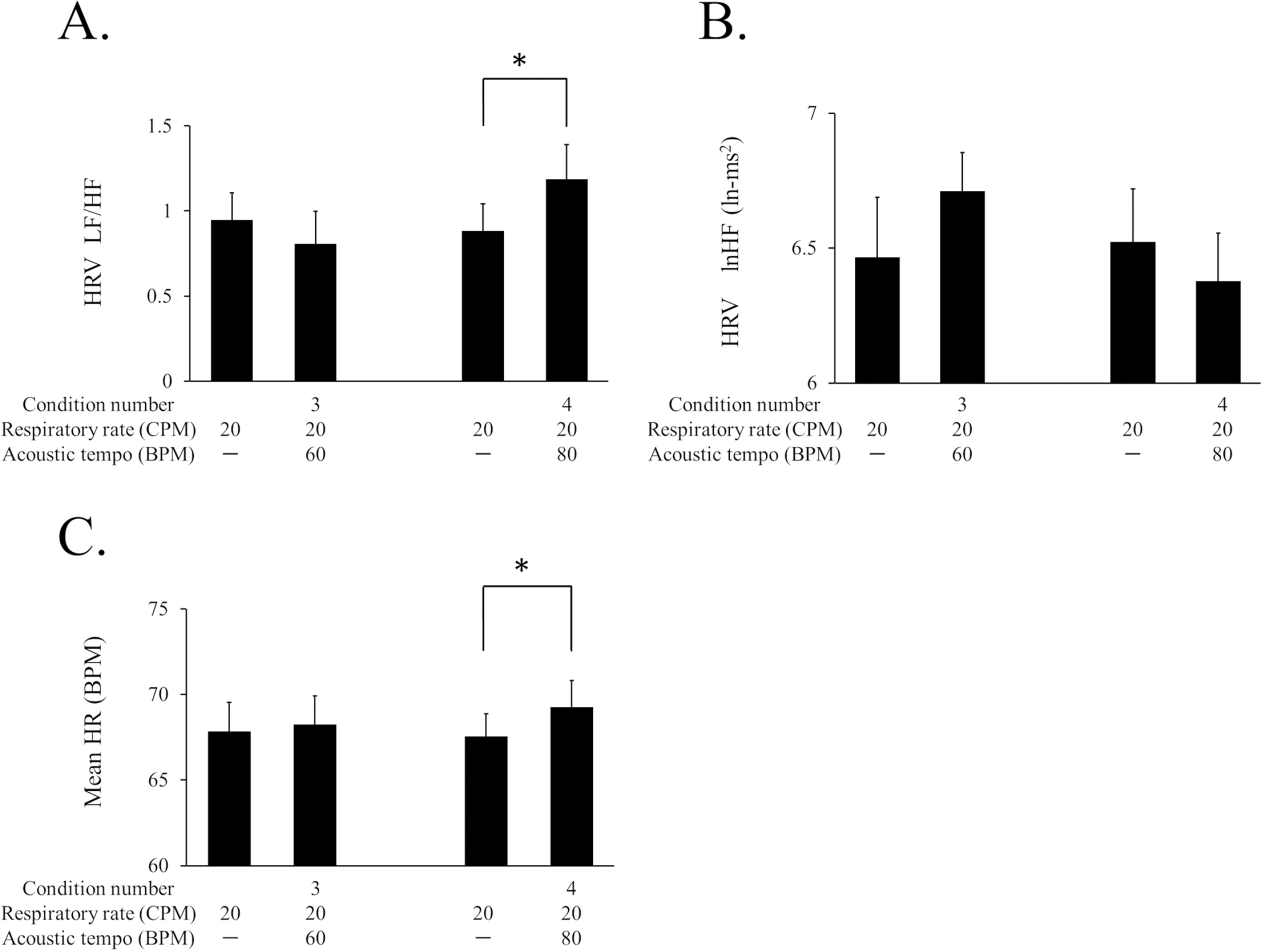
The HRV data and the mean HR of each session in Experiment 3. Conditions 3 and 4 were compared with the baseline of each condition represented on the left of each graph. A: LF/HF of the HRV of each condition. B: lnHF of the HRV of each condition. C: The mean HR of each condition. The bar graphs and error bars represent the mean±SEM. The statistical significance is indicated as *p < .05.

The effect of the acoustic tempo on autonomic nerve activity at the faster respiratory rate was measured as a change in HRV from the baseline to condition 3 or 4. LF/HF increased significantly with condition 4. A Wilcoxon sign rank test demonstrated that the LF/HF of condition 4 was significantly larger than the baseline value, *T* (16) = 33, *r* = 0.499, *p* < .05 (Fig 6A). There was no significant change in lnHF for condition 3 or 4 (Fig 6B).

The mean HR of each condition was also calculated to confirm the increase in the mean HR of condition 4 (Fig 6C). The HR change was evaluated by averaging the data from t = 55 to t = 295 as well as HRV. A paired *t* test revealed that the mean HR of condition 4 was significantly larger than the baseline value, *t* (16) = 2.65, Cohen’s *d* = 0.29, *p* < 0.01.

## Discussion

The current study showed that the sympathetic nerve is activated by sound stimuli with a fast tempo when the respiratory rate is also fast. Although the mean HR was not increased by sound stimuli when the acoustic tempo or respiratory rate was slow (60 BPM for acoustic tempo, 15 CPM for respiratory rate), sound stimuli with a faster respiratory rate–faster acoustic tempo combination (20 CPM– 80 BPM) could increase the mean HR. Under this condition, the LF/HF of the HRV increased while the HF was unaffected, indicating that this increase in the mean HR is caused by the enhancement of sympathetic nerve activity. These results suggest that the enhancement of the sympathetic tone requires not only a fast acoustic tempo but also a fast respiratory rate when listening to music.

Additionally, we observed that an increase in the mean HR was induced by sound stimuli with an acoustic tempo of 78 or 82 BPM as well as that of 80 BPM when the respiratory rate was controlled at 20 CPM (Fig 5). It is possible that the respiratory frequency stay in phase with acoustic tempo by the entrainment of respiratory cycle to acoustic tempo even if the acoustic tempo was 78 or 82 BPM. However, the respiratory frequency stays in phase with metronome cycle (S1 Fig). Therefore, as regards the music-induced activation of the sympathetic nerve it is not essential that the respiratory frequency stay exactly in phase with the acoustic tempo.

Many studies have investigated the effects of music on human beings by performing brain imaging [16, 17], measuring the respiratory rate [1, 18] or measuring autonomic nerve activity [3, 7]. However, there have been few studies focusing on different individual musical parameters. Bernardi et al. (2006) reported that music at a fast tempo caused an increase in the respiratory rate, mean HR and the LF/HF of HRV. Their study suggested that musical tempo has a greater effect than style of music or a person’s musical preference. These results implied that musical tempo affects the sympathetic center and is related to the respiratory center. Bernardi’s group have also reported the effect of breathing rate on the ANS and shown that a slower breathing rate suppresses the chemoreflex response to both hypoxia and hypercapnia and enhances baroreflex sensitivity [8, 9]. However, they did not consider the effect of the interaction between acoustic tempo and respiratory rate. Our findings suggest a novel neural mechanism existing between auditory, respiratory and sympathetic nervous systems.

The RVLM is one of the primary regulators of the sympathetic nervous system as regards vasoconstriction and arterial pressure, indicating that it is the regulator of the sympathetic nerve that controls cardiac activity. The RVLM receives direct or indirect neuronal inputs from the auditory and respiratory systems. The auditory system is indirectly related to the RVLM through the amygdala. Neurons that consistently respond to repeated auditory stimuli are located not only on the medial geniculate body (MGB) but also on the lateral amygdala (LA) [19], suggesting that there are neuronal connections between the MGB and LA. Additionally, the auditory cortex (AC) has neuronal projections to the LA [20]. The LA projects to the central nucleus of the amygdala (CeA), and then nucleus tractus solitarius (NTS) receive the inhibitory input, and the caudal ventrolateral medulla (CVLM) receives the excitatory input from the CeA. This inhibition of the NTS reduces the inhibitory inputs from the CVLM to the RVLM, which then activates neurons in the RVLM, leading to an increase in sympathetic outflow [21, 22]. Therefore, the change in auditory stimuli indirectly affects the RVLM through the amygdala.

On the other hand, there is at least some sympatho-respiratory coupling between the sympathetic nervous system and the respiratory system in the medulla [23–27]. The center of respiratory rhythmic activity is located within the ventral respiratory column (VRC) of the medulla, and generates a respiratory rhythm and pattern [28]. Beakey et al. (2010) suggested that there are inhibitory connections from the Bötzinger complex (part of the VRC) to the RVLM and excitatory connections from the pons to the RVLM [29]. Additionally, Moraes et al. observed that the phrenic nerve is synchronized with the thoracic sympathetic nerve and RVLM activity, indicating that respiratory oscillation is synchronized with the oscillation of sympathetic activity [30].

**Fig 7:**
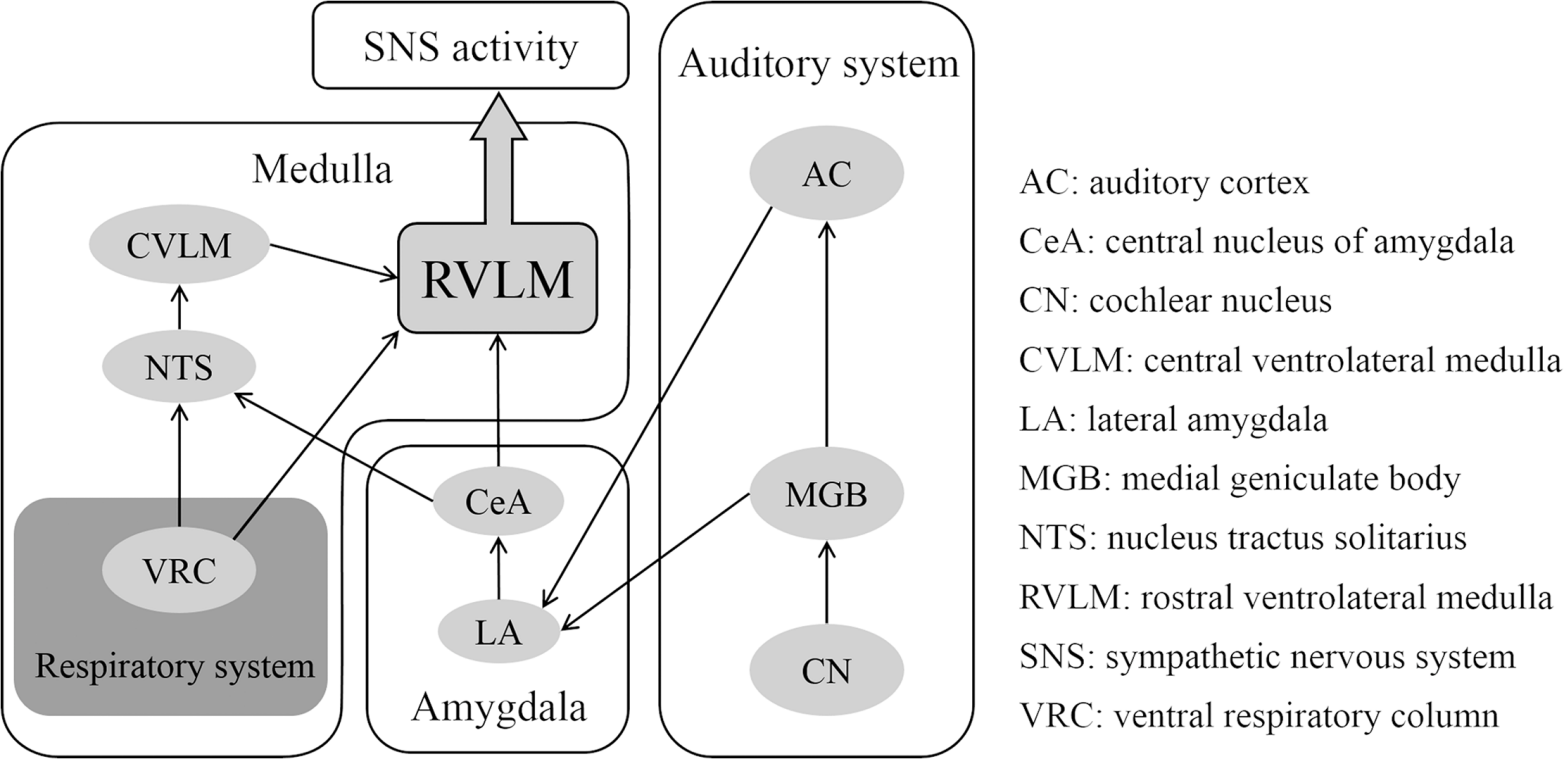
Schematic view of the neural mechanism of the auditory system, amygdala, respiratory system and medulla. The lateral amygdala receives neuronal input from the auditory thalamus (medial geniculate body) and auditory cortex (primarily association areas). The central amygdala projects to the nucleus tractus solitarius and rostral ventrolateral medulla. The rostral ventrolateral medulla is the primary regulators of the sympathetic nervous system as regards vasoconstriction and arterial pressure. On the other hand, the ventral respiratory column, which includes the Bötzinger complex, which is considered a major source of rhythmic inspiratory activity, projects to the NTS and RVLM. The solid arrows represent neuronal connections.

Thus, the RVLM, which is the primary regulator of sympathetic nerve activity related to blood pressure, is affected by both the auditory and respiratory systems. Fig 7 shows schematic views of the neural connectivity mechanism of the auditory system, amygdala, respiratory system and medulla as described above.

We speculate that the neural mechanism noted above explains our results. In the current study, the change in respiratory rate alone had no effect on the mean HR. Stark et al. (2000) measured the effects of four respiratory frequencies (0.15, 0.20, 0.25, 0.30 Hz) on the mean HR and suggested that a respiratory frequency in the 0.15–0.30 Hz range cannot affect the mean HR [15].

We observed that the mean HR remained unchanged when a person listened to sound with a fast tempo (80 BPM) when the respiratory rate was controlled at 15 CPM, although the mean HR increased when the respiratory rate was controlled at 20 CPM. These results suggest that the effect of acoustic tempo on the sympathetic tone is modulated by the respiratory system and sympatho-respiratory coupling plays the critical role of “enhancer” for a music-induced sympathetic tone. Listening to sounds with a fast tempo under fast respiratory rate conditions could cause an increase in the RVLM firing rate. This interaction between the auditory and respiratory systems is consistent with the results of a previous study, suggesting that a respiratory-driven sympathetic tone works as a gate for a sympathetic tone induced by sound stimuli [10].

One might suspect that visual stimulation would influence autonomic nervous activity, but in this study, the effects of visual stimulation could be ignored. We performed an additional experiment to confirm that the effects of visual stimulation are small. In the additional experiment, the respiratory rates of the participants were controlled by sound stimuli, in the same way as in our other experiments. In the result, a paired *t*-test demonstrated that the mean HR of condition 4 was significantly larger than the mean HR of condition 3 (S2 Fig). Therefore, we were able to observe the same effects of the respiratory rate and acoustic stimulus even if the ANS was not influenced by a visual stimulus.

On the other hand, one might suspect that the voluntary control of respiration could influence autonomic nervous activity, but in this study, the effects of voluntarily controlling the respiration could be inclusion. As mentioned below, we only observed the change from the baseline and were able to observe the effects of the respiratory rate and acoustic stimulus even if the ANS was already affected by voluntary respiration control.

In this study, we discovered that the combination of respiratory rate and acoustic tempo is important as regards the change in heart rate induced by sympathetic nerve activity. Since the only musical characteristic that we changed was the acoustic tempo, we could clarify the effects of musical tempo on human beings discretely. We conclude that tempo is one of the most important characteristics of music, and the effect of acoustic tempo on the sympathetic tone is modulated by the respiratory system. This discovery may be useful in such medical fields as music therapy.

## Supporting Information

S1 Fig The time series of the respiratory rates of each condition in Experiment 1 and 2. The vertical axis indicates the respiratory rate. The points represent the averaged respiratory rates of all the participants. The graphs represent the respiratory rates of the condition 1 (Fig A), condition 2 (Fig B), condition 3 (Fig C), condition 4 (Fig D) in Experiment 1, and condition 4 (Fig E), condition 5 (Fig F), condition 6 (Fig G) in Experiment 2. We applied 1-min window for statistical analysis and compared with metronome cycles. The respiratory rates averaged by 1-min window were analyzed with a two-factor repeated measures analysis of variance (ANOVA) [condition (2) x time (5)] as within-subjects factors. No significant change in respiratory rate with respect to a metronome was observed.
(TIF)

S2 Fig The mean HR in additional experiment. The vertical axis indicates the mean HR of condition 1 and 4 when the respiratory rates of the participants were controlled by sound stimuli, in the same cycles as in same condition number. In additional experiment, the sound stimuli were same as our other experiments. The bar graphs and error bars represent the mean±SEM. Statistical significance is indicated as *p < .05.
(TIF)
