# Supplementary figures and images for: Sympathetic Tone Induced by High Acoustic Tempo Requires Fast Respiration
Source: PLoS One. 2015 Aug 18;10(8):e0135589. doi: 10.1371/journal.pone.0135589 (PMC4540583; doi:10.1371/journal.pone.0135589)

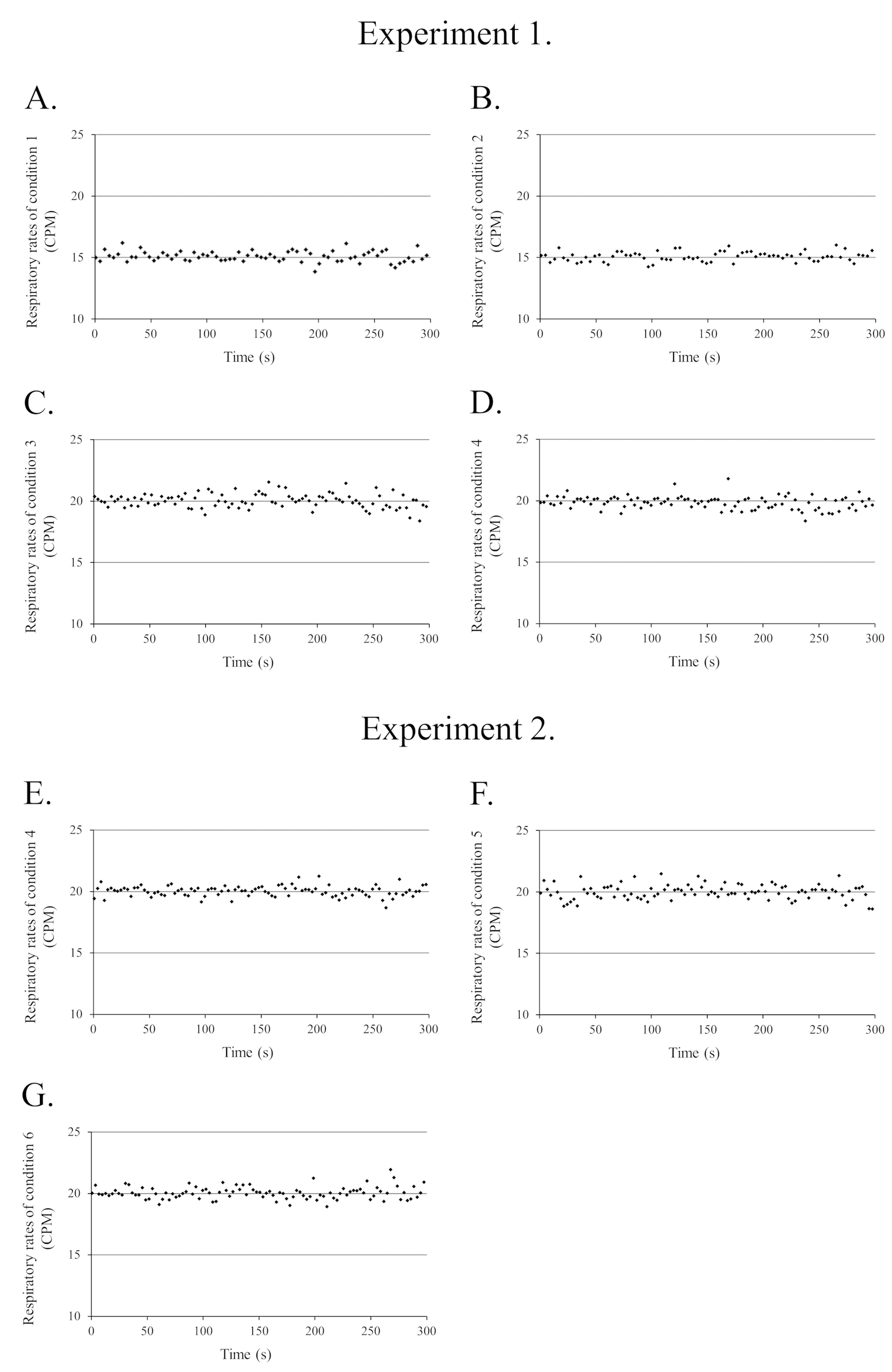

Supplement: S1 Fig — The vertical axis indicates the respiratory rate. The points represent the averaged respiratory rates of all the participants. The graphs represent the respiratory rates of the condition 1 (Fig A), condition 2 (Fig B), condition 3 (Fig C), condition 4 (Fig D) in Experiment 1, and condition 4 (Fig E), condition 5 (Fig F), condition 6 (Fig G) in Experiment 2. We applied 1-min window for statistical analysis and compared with metronome cycles. The respiratory rates averaged by 1-min window were analyzed with a two-factor repeated measures analysis of variance (ANOVA) [condition (2) x time (5)] as within-subjects factors. No significant change in respiratory rate with respect to a metronome was observed. (TIF) [file pone.0135589.s001.tif]

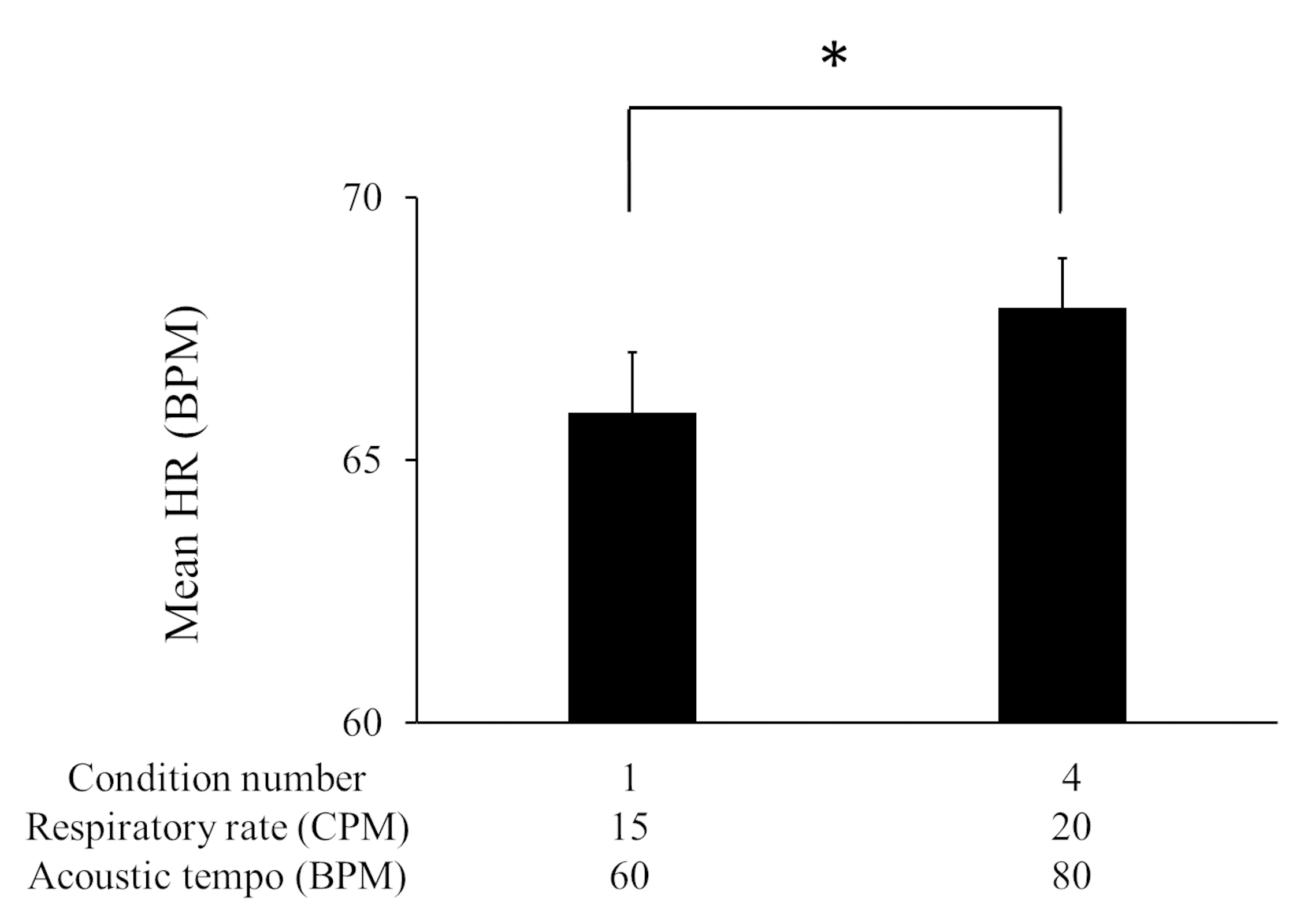

Supplement: S2 Fig — The vertical axis indicates the mean HR of condition 1 and 4 when the respiratory rates of the participants were controlled by sound stimuli, in the same cycles as in same condition number. In additional experiment, the sound stimuli were same as our other experiments. The bar graphs and error bars represent the mean±SEM. Statistical significance is indicated as *p < .05. (TIF) [file pone.0135589.s002.tif]
